# Losing Meaning: Philosophical Reflections on Neural Interventions and their Influence on Narrative Identity

**DOI:** 10.1007/s12152-021-09469-5

**Published:** 2021-05-15

**Authors:** Muriel Leuenberger

**Affiliations:** Department of Arts, Media and Philosophy, University of Basel, Steinengraben 5, 4051 Basel, Switzerland

**Keywords:** Narrative identity, Self, Meaning, Deep brain stimulation, Psychopharmaceuticals, Psychedelics, Prozac, Ritalin

## Abstract

The profound changes in personality, mood, and other features of the self that neural interventions can induce can be disconcerting to patients, their families, and caregivers. In the neuroethical debate, these concerns are often addressed in the context of possible threats to the narrative self. In this paper, I argue that it is necessary to consider a dimension of impacts on the narrative self which has so far been neglected: neural interventions can lead to a loss of meaning of actions, feelings, beliefs, and other intentional elements of our self-narratives. To uphold the coherence of the self-narrative, the changes induced by neural interventions need to be accounted for through explanations in intentional or biochemical terms. However, only an explanation including intentional states delivers the content to directly ascribe personal meaning, i.e., subjective value to events. Neural interventions can deprive events of meaning because they may favor a predominantly biochemical account. A loss of meaning is not inherently negative but it can be problematic, particularly if events are affected one was not prepared or willing to have stripped of meaning. The paper further examines what it is about neural interventions that impacts meaning by analyzing different methods. To which degree the pull towards a biochemical view occurs depends on the characteristics of the neural intervention. By comparing Deep Brain Stimulation, Prozac, Ritalin, psychedelics, and psychotherapy, the paper identifies some main factors: the rate of change, the transparency of the causal chain, the involvement of the patient, and the presence of an acute phenomenological experience.

## Introduction

Neural interventions, such as brain implants or psychopharmaceuticals, can lead to profound changes in personality, mood, and other features of the self. The impact of neural interventions on the narrative self, which understands the self as constituted by a self-narrative, has raised concerns and become a debated topic in neuroethics, especially in the context of Deep Brain Stimulation (DBS) [1–8]. DBS makes use of a surgically implanted electrode that stimulates targeted brain areas to treat a growing set of motor disorders and mental health conditions. The neuroethical debate on DBS emerged after studies reported that some patients experienced far-reaching and sometimes sudden personality changes and feelings of alienation [9, 10]. The prospect of changing personality through a targeted electric stimulation has raised concerns about identity, authenticity, and related concepts, including worries about the narrative self [1–8, 11–14]. A similar debate followed the increased use of psychopharmaceuticals, in particular the antidepressant Prozac, in the 1990s and early 2000s [15–17].

The self-narrative can be understood as an internalized, evolving story recounting one’s life-events from a personal perspective, reflecting character traits, goals, and values. Through a self-narrative, humans organize their lives, contextualize single episodes, and ascribe personal meaning and significance to events. Whether someone changes because they were influenced by a new circle of friends, because they took an antidepressant, or because they underwent DBS has important implications for the self-narrative. Possible personality changes induced by neural interventions have raised two main concerns regarding narrative identity: disruptions to the coherence of the self-narrative [7, 18] and possible threats to autonomy and agency in cases where a neural intervention replaces informed and rational choices or disrupts narrative self-revision [1, 4, 8]. In this paper, I argue that it is necessary to consider a further dimension of impacts on the narrative self: neural interventions can lead to a loss of meaning of actions, feelings, beliefs, and other intentional elements of our self-narratives. I agree that neural interventions can threaten the coherence of the self-narrative but other transformative experiences can be equally disruptive to the narrative. What sets neural interventions apart is not their impact on coherence but on meaning. Thus, the argument is in agreement with the observation that for most people, changing through direct neural interventions seems more problematic than through other means. The concerns which have been raised regarding autonomy and agency are based on a relational concept of narrative identity. I do agree that these concerns point towards important and valid phenomena regarding the impact of neural interventions. However, I adopt an internalized version of narrative identity and understand these phenomena as having only an indirect impact on identity. My argument should nonetheless be compatible with a relational account of narrative identity. Besides showing that direct neural interventions can have a particular effect on meaning, I discuss what it is about the means of neural intervention that impacts meaning by comparing different methods. Thus, I argue that the reasons why the means of a neural intervention matter go beyond the distinction between passive and active interventions [3].

The paper can be summarized as follows. Second section: Through narratives, we give meaning to the events of our lives, be it single actions, episodes stretching over longer time frames, or recurring patterns. It is possible to account for actions, beliefs, moods, or emotions via intentional states, biochemical processes, or a combination of both. However, only an explanation referring to intentional states directly ascribes personal meaning. The second part of this section explores the ethical significance of a loss of meaning. Third section: Even though it is possible to explain the change induced by neural interventions at least partially through intentional states, neural interventions can favor a predominantly biochemical perspective, leading to a loss of meaning. How strong the pull towards the biochemical perspective is, depends on the specific method. Therefore, I discuss and compare four different means of intervention: Deep Brain Stimulation, Prozac, Ritalin, and psychedelics. These examples are meant to represent paradigmatic ways of inducing self-change. A brief discussion of psychotherapy will serve as an example contrasting direct neural interventions. The effect of many other kinds of neural interventions should be derivable from the discussion of these cases. The main factors influencing which perspective is favored are the rate of change, the transparency of the causal chain, the involvement of the patient, and whether the neural intervention causes an acute phenomenological experience. Fourth section: In the conclusion, I summarize and give a brief outlook on implications for treatment choice and further research.

## Meaning through Narratives

Theories of the narrative self have gained popularity and advanced various areas of the philosophical discourse. According to the narrative self view, humans integrate their experiences into a linear, internalized, and evolving story, which constitutes the self.^1^ Single episodes are connected, ordered, and contextualized through the self-narrative. This temporally extended self-representation shapes and organizes one’s experience. The present moment is understood and experienced through its position in the narrative of one’s life.^2^ This does not mean one has to consciously tell one’s life-story to oneself or others. It is a largely implicit understanding of oneself as being at a certain point in an unfolding life. You have a certain history and an anticipated future, which not only influence who you are now and who you are going to be but also how you experience and interpret the present moment. For someone who has always been poor, walking through a grocery store is a different experience compared to someone who never had to worry about money or even for someone who thinks that he will be wealthy in a few years [21]. The narrative self or narrative identity, constituted by the self-narrative, addresses the so-called characterization question: which actions, experiences, beliefs, and other characteristics are we to attribute to a person? What makes me *me*? [21].

Besides connecting selfhood to a self-narrative, narrative self views argue that to ascribe meaning to an event one has to understand it in narrative terms, in contrast to a naturalistic, reductionistic description. The events of our lives gain their personal meaning through their position in the self-narrative [21–24]. By personal meaning, I refer to the subjective value an event has, its personal significance, as opposed to functional or semantic meaning. By integrating events into a self-narrative, they receive the necessary context of intentions, beliefs, personal history, and institutional settings, i.e., of intentionality,^3^ to be meaningful. What it means for a person to win a tennis tournament can only be understood by realizing that she, for instance, trained her whole life for this and hopes to become a professional player. The short- and long-term causally effective intentional states and their connection to each other constitute the meaning of an event. Thus, an event derives its meaning from the causal and temporal ordering of the underlying intentions and beliefs, i.e., from the narrative it is situated in [23]. Naturally, it is possible to account for events in biochemical or physical terms instead of intentional ones—in terms of the *nomological realm of law* [25] instead of the *logical space of reasons* [26]. However, to understand the meaning of an action, a belief, or a feeling, knowing the underlying biochemical process, such as the neural activation pattern and the muscle contractions, does not suffice. The intentional, diachronic perspective is necessary to ascribe subjective value (i.e., meaning) to an event. Coherence is a further precondition for the ascription of meaning. Only if short- and long-term causally effective intentional states are ordered with reasonable coherence they take the form of a self-narrative. Only through a coherent narrative we can make sense of them and ascribe meaning.

Of course, not everything in our lives is meaningful. Events lack meaning if they are largely irrelevant to the bigger picture, such as someone scratching his head, or if they are non-intentional. A car accident, for instance, is non-intentional because it is not *about* something, representing something, or manifesting a value or an intention. However, such coincidental events can make us seek new values, abandon previous ones, or make our goals unattainable. These non-intentional events do play a role in meaning-generating self-narratives but they do not generate the meaning themselves. They can gain meaning indirectly through their connection to intentional states. They reshape the framework in which we act and can take or give support to our beliefs and intentions. A paralyzing accident, for instance, while having a big impact on a person’s life, is only meaningful insofar as it impacts and reshapes other, meaningful events. Some people do ascribe more meaning to coincidences and accidents and see them as somehow meant to be. But in doing so, they see them as manifesting the intentions of a higher being or the universe. Even in these cases, meaning is ascribed via narratively ordered and connected intentional states. Similarly, in books, we interpret even a coincidence as meaningful because it results from the author’s intentions.

This lack of meaning can apply to one’s own behavior. One’s perspective on a certain behavior can shift from a meaningful, intentional action to a purely biochemical process. Viewing ourselves from the latter perspective is not unusual. For instance, being hungry can sometimes serve as an explanation for angry behavior. Such biochemical explanations can make sense and be helpful. If a person knows she tends to be “hangry” (to be irritated when she is hungry) she does not try to understand her anger as a result of her character or another person’s behavior. A biochemical explanation can grant relief from the search for an intentional explanation of behavior by providing another way to uphold the coherence of the narrative, albeit one in which behavior loses its personal meaning. Of course, the two ways of accounting for behavior can also be mixed.

It has been claimed that an explanation of self-change through psychopharmaceuticals leaves a gap in the narrative, diminishing its coherence and leading to *hyponarrativity*—the inability to foster construing a self-narrative [18].^4^ Hoffman suggests the following as an example of pharmaceutically induced hyponarrativity: *“The reason I came to believe that the world is worth living in after all is because more of my serotonin transporters were blocked.”* However, if we understand coherence as intelligibility, as a measure of whether the narrative makes sense and is comprehensible, such a causal view on behavior, a belief, or a feeling can be coherent. They are still accounted for and explained without leaving a gap. The effect of a shift to a biochemical perspective is not a lack of coherence but a lack of meaning.

### The Ethical Significance of a Loss of Meaning

After establishing how a biochemical perspective can lead to a lack of meaning we may turn to its ethical significance. Why should we care if an event is no longer meaningful? One reason is that meaningful interactions, feelings, beliefs, and actions can give one’s life direction, purpose, and a sense of fulfillment. A life understood as meaningful contains many meaningless experiences. But it can be unsettling if an event, a feeling, an action, or a belief which used to carry meaning loses it. Another reason is grounded in the fact that in a narrative view of the self, the meaning we ascribe through the self-narrative is defining ourselves. Either I am the person that experienced deep sorrow in the face of the sorry state of the world or I am the person whose neural network fired in a way that made me experience sorrow. Both descriptions may be correct but which one I take and integrate into my self-narrative shapes how I see myself, how I organize my experiences, and ultimately who I am. It can affect a specific aspect of the self-narrative or it can have a larger impact on the narrative and thereby on one’s identity as a whole, in case central elements of one’s self are affected. A shift to a biochemical perspective may not only lead to distressing instances of loss of meaning but it impacts who I am as a person. This is not problematic in every case and it can even be liberating to no longer ascribe meaning to certain actions or feelings (as in the hangry example). But such a loss of meaning and the connected changes in one’s identity can be disturbing for the affected individual as well as their friends and family. For some, it is possible to find meaning in other elements of one’s life but in some cases, it could lead to a crisis of purpose or identity.

In the following, I want to shed some light on circumstances that can make a loss of meaning more problematic than, for instance, the hangry example and which will be important for the discussion on neural interventions:

1. A loss of meaning can concern events and characteristics one was not ready and willing to have stripped of meaning. This seems to have occurred to Sam, a case discussed by Kramer (1997). After treating his depression with Prozac, Sam lost his former rough edges and his interest in pornographic videos. “He experienced this change as a loss. The style he had nurtured and defended for years now seemed not a part of him but an illness.” [15] Sam not only lost interests and traits he cared about, but was also led to question their genuineness and meaning in his past. Similarly, a moment that connected a person with a loved one could eventually appear to have been directed by biochemical processes and not meaningful intentional states. Thereby, the loss of meaning can also affect the self-narratives of other people who shared those moments.
2. A loss of meaning can be harder to deal with if it happens suddenly and without sufficient preparation. In Sam’s case, the effectiveness of Prozac may have led him to change the perspective on his interests and traits quite abruptly. Suddenly, an intentional view became implausible. If a biologized perspective is adopted only gradually, the individual has time to slowly adjust the self-narrative, to redefine himself via other traits, and interests and to find meaning and self-definition elsewhere.
3. An involuntary and uncontrolled shift in perspective could lead someone to question the accuracy of her self-narrative, and thus her identity, in general. It may raise doubts about the genuineness of her views, feelings, and actions: *if those actions/traits are only the results of biochemical reactions maybe loving my husband is too*. This can be disturbing and lead to a feeling of lost control or an identity crisis. Moreover, we can be reminded of the possibility that the *logical space of reasons* could be reducible to the *nomological realm of law*.^5^
4. Shifting to a biochemical perspective on actions, feelings, or beliefs often, though not necessarily, means seeing them as an external influence and not part of oneself. In the hangry case, it may not be overly troublesome to distance oneself from these moods and actions. But it can be troublesome to understand central parts of one’s identity as a mere external influence. What is left may not seem like a particularly rich and well-defined self. Sam saw the traits and interests changed by Prozac as expressing who he is as a person. The use of Prozac redefined what is essential and what is contingent about Sam’s personality. It can be difficult to adjust to the externalization of something deemed integral to one’s identity.
5. Lastly, conflict can arise between individuals and their families and friends in case they disagree on which perspective best fits the situation. A person may understand his characteristics as expressions of his own beliefs and intentions whereas his social environment sees them as purely biochemical reactions, expressions of a chemical substance or electric stimulation, and vice versa.

Taking a biochemical perspective on one’s doings has further implications beyond a loss of meaning. It can affect matters of control. Behavior understood as governed by a biochemical process, as in the case of the hangry person, is not under one’s direct control. Intentional states can only influence it indirectly. In regular circumstances, our actions are driven by intentional states which make sense to us in the light of our self-narratives, which have a meaningful connection to our past and to other things we care about. Even when our intentional states change very suddenly, they are accounted for through the narrative. For instance, after a traumatic experience, someone could suddenly abandon long-held plans and make new ones but she could still understand that she acquired them because of what she experienced. They are not a mystery to her. In contrast, behavior understood as governed by biochemical processes is not accounted for by past experiences or intentional states. It can seem to come out of nowhere, especially if experienced for the first time. Thereby, such behavior can provoke a feeling of lost control.

In a similar vein, it is harder to identify with events that are not meaningfully integrated into the self-narrative. It is possible to view what a person does because he is hangry as not representative of who he is. This anger is not something he *is* in a meaningful way but something he *suffers*. Thereby, the behavior is externalized and not fully self-owned. Especially if such a behavior is experienced for the first time, it may be alienating. With time, intentions and beliefs can adapt to biological patterns and restrictions. Moreover, it becomes possible to anticipate the behavior and develop strategies to cope with it and to exert indirect control. Thereby, this behavior falls back into one’s area of responsibility and, in a broad sense, into the *space of reasons*. This may facilitate identifying with such behavior but the possibility of externalization remains. Thus, a loss of meaning can bring along a loss of identification.

Loss of meaning further impacts properties and concepts which relate to the self-narrative, such as authenticity, autonomy, identity, and responsibility. For example, self-knowledge plays an important role in the concept of authenticity. Through a neural intervention, one’s self-knowledge can be questioned if one’s perspective on an event suddenly shifts. If I was wrong in thinking my depressed thoughts were meaningful, I might be wrong about other parts of my identity. Furthermore, authenticity requires a well-defined self, as I have argued elswhere [27]. Ascribing more characteristics, feelings, and actions to biochemical influences can lead to a lack of self-definition. People are defined by the meaningful actions they perform or feelings they experience and not primarily by the biochemical influence they are subject to. What would *I* have done if the implant, the drug, or the disease did not make me take this action? However, the relationship between loss of meaning and properties and concepts like authenticity, autonomy, identity, and responsibility is complex and beyond the scope of this paper.

## Neural Interventions and Loss of Meaning

In the following, I argue that neural interventions can lead to a shift from viewing events, actions, beliefs, or feelings as meaningful responses to meaningful situations, to a predominantly biochemical view, which deprives them of meaning.^6^ Moreover, four different kinds of paradigmatic means for neural intervention (Deep Brain Stimulation, Prozac, Ritalin, and psychedelics) are compared to analyze which aspects of the intervention can promote this loss of meaning. As an example of an indirect neural intervention, psychotherapy is briefly discussed. Of course, how a neural intervention impacts the self-narrative and meaning depends on the individual case and the specific context. What is discussed in the following is but one element among many which play a role.^7^ Despite the case-dependency, the following can explain an important impact neural interventions can have on the self-narrative as well as common worries regarding neural interventions.

Before comparing the various means of neural intervention, I want to address the difference between direct and indirect interventions [3, 31, 32]. A direct intervention alters mental states on a physical level, following laws of nature, through, for instance, a psychoactive chemical substance or psychosurgery. The brain is targeted directly, bypassing the mental faculties of the patient. In an indirect intervention, one’s mental faculties can weigh in on the course of self-change. Usually, we are influenced by indirect means, for example, by reading an article, talking to a friend, or experiencing a traumatic event. Of course, indirect interventions also alter the brain. But they go through the “usual” pathway, designed to process the information. In the case of indirect interventions, self-change is mediated by internal processes, involving meaningfully connected intentional states.

It has been argued that the biographical disruption that, for example, Michael J. Fox experienced through Parkinson’s Disease (PD) is not a threat to his identity and that this disruption is comparable to disruptions caused by DBS [1]. I agree that the disruptive potential is comparable. But the crucial difference between a transformation through PD and a direct intervention like DBS lies not in the degree to which a life changes but in the way the change was induced. A disease like PD can force individuals to change and to adjust their life-plans. However, the direction of the change is guided by both the intentions, beliefs, and characteristics of the individual as well as the constraints and opportunities of the new situation. This allows for a view on the change as a meaningful response to a difficult situation. Of course, PD directly affects the brain. But many effects PD has had on the life of Michael J. Fox are indirect. Internal processes, reflection, and consideration led him to launch a foundation for PD research and write books. The way this disease transforms him connects to who he is, where he came from, and what is meaningful to him. Therefore, the change makes sense on a narrative level, based on intentional states (this does not exclude that it could in principle be explained or even predicted through a purely biochemical, reductionistic account).

In the case of direct interventions, two attributes can foster a perspective which deprives self-change of meaning. First, direct interventions are, in an important sense, synchronic. They work independently of the patient’s past and are not connected to previous experiences or intentional states, unlike indirect interventions. Direct interventions implement change depending on physical interactions between, for instance, the implant and neurons and not one’s position in the self-narrative. The biochemical reactions are independent of my plans for my future or my social support system whereas indirect interventions revolve around them. Of course, one’s past has influenced the brain structures direct interventions manipulate. But a brain implant does not pick up on the patient’s past experiences in the same way as an indirect intervention. Second, direct interventions do not provide situational content that explains the direction of the self-change. If I change my values after reading a book the *content* of the book helps to explain why I ended up with these new values and not others. The same goes for changes that occurred because the individual faces a new situation, such as after a paralyzing accident. There is *content* to the situation which is guiding the direction of change in a way one can generally follow. In the case of direct interventions, taking a pill or undergoing brain-surgery explains the change on a biochemical level but it does not deliver the content for a meaningful, narrative explanation. The situation which leads to the change is not sufficiently content-rich to deliver a meaningful account of how the individual ended up with exactly those changes.

This loss of meaning through direct neural interventions can however be mitigated. For example, a person choosing to use a neural intervention with the precise purpose of changing her mood or behavior by direct means, fully expecting it to do so and recognizing it for what it is when it happens will find these changes intelligible and meaningful in light of her intentions and wider life project.^8^ The change brought about by the neural intervention is not meaningful in and of itself since it is an unintentional, biochemical process. But it is deeply embedded in meaningful choices, beliefs, goals, and intentions which indirectly provide meaning. They play a part in an overarchingly meaningful episode without contributing to the meaning themselves. Moreover, even if patients experience an initial loss of meaning regarding some characteristics, feelings, or actions after treatment, these changed parts of themselves may eventually regain meaning. New characteristics can be integrated into new stories, relationships, and meaningful moments. They may become familiar and integral parts of one’s life. With time, the biochemical perspective on them can fade and it is possible to identify with these characteristics, feelings, or actions. Thereby, new attributes can become fully integrated into the self-narrative in a meaningful way. Below, I discuss how the different methods of neural intervention allow for further strategies to avoid or reduce a loss of meaning.

Changing aspects of oneself that were not intentional in the first place, for instance when a neural intervention reduces tremor, does not lead to a loss of meaning. With regard to mental disorders, however, patients can be in a state of uncertainty about the nature of their behavior. Several issues complicate the matter: it is not always clear-cut what is caused by the disease and what is non-pathological behavior; it can be unclear whether a mental condition should best be understood and treated from a biochemical perspective as a neurological disorder, or from a psychological perspective; and it is possible to view a mental disorder as manipulating one’s intentional states or as a manifestation of them. These different perspectives can impact the loss of meaning through treatment as well as how meaningful the behavior, moods, feelings, or beliefs are seen independently of treatment. Many patients already experienced substantial change through their disorder before using a neural intervention. For instance, DBS is most commonly used to treat patients with PD, which can lead to severe neuropsychiatric disturbances [33]. Besides the indirect influences discussed in the example of Michael J. Fox, PD can also directly influence the mind. If a mental condition is treated which plays the role of a direct, biochemical influence in the person’s self-narrative, the neural intervention can be experienced as a restoration, as a neutralization of the biochemical influence of the disorder. In this sense, the individual may even understand her actions, feelings, or beliefs as more intentional and meaningful than before the stimulation.^9^

### Deep Brain Stimulation

DBS is a neurosurgical procedure in which implanted electrodes stimulate targeted brain areas. The low current emitted by the electrodes is regulated by a stimulator implanted in the chest area. A remote control can be used to turn the device on and off and to adjust settings. DBS is used for the treatment of Parkinson’s Disease, movement disorders, epilepsy, and at an experimental stage for mental conditions such as Obsessive–Compulsive Disorder, Anorexia Nervosa, and depression. DBS has sparked a debate in neuroethics because of its potential to induce changes in personality, identity, autonomy, authenticity, agency, and self [2, 7, 12–14, 35]. After stimulation, some patients reported feelings of alienation, increased impulsiveness, loss of vitality, changes in mood and libido, or that they were “feeling like a robot” [9, 10, 36–38]. The extent to which DBS causes unintended changes in personality, identity, autonomy, authenticity, agency, and self is a matter of debate [35, 39, 40]. More empirical research is warranted to assess how prevalent such changes are and whether they are caused by DBS. However, the argument of this paper also applies to cases in which changes of personality are the aim of the treatment. For example, when DBS is used to treat depression or other mental health conditions. I use DBS as an exemplary case of a technological neural intervention that can induce fast, far-reaching changes in a patient who is largely passive.^10^ To illustrate the possible effects of DBS and the potential shift from a meaningful view on an event to a biochemical one, we may consider an example. Helmut Dubiel received DBS to treat PD but as a side-effect, he developed depression. The following describes his experience when the device settings were adjusted:

*Within one second, a small change in the voltage and a simple polarity reversal of the electrodes in my head improves the massive depression I had been experiencing for a year. I was both fascinated and frightened that the depression fell away from me just like that, as if an iron band around my soul had snapped. The very ease of the process intrigued me. The press of a button, confirmed through a barely audible digital beep and supported by a tiny LED, and my overcast skies instantly cleared. The friends I called thought I had just fallen in love, that’s how happy I must have sounded. Frightening and also somehow humiliating was the banality of the process. I had felt the weight of the world in the innumerable sorrowful tales that had gone through my head that year. Simply to wipe it all away at the push of a button seemed almost frivolous.* [41]

This case shows how the perspective on his depression, as emerging from sad tales, as feelings that are to some degree reasonable and connected to one’s intentions, values, and motives, shifts to a physical perspective. The fact that these sorrowful tales can be wiped away by a well-placed electrical stimulus rids them of their depth and meaning. The depression turns from a meaningful response to a meaningful situation to a defective neuronal state. If a state of depression disappears at the push of a button, there do not seem to be any underlying reasons for it rooted in who I am and what I went through.

Besides the fact that DBS is a direct intervention, there are three main reasons why it can lead to a loss of meaning.

1. As shown in Dubiel’s example, the induced changes can be severe and instant. Several studies report similarly fast and far-reaching changes [36, 37, 42–44]. The immediate response to the activation or adjustment of the device leaves no room for interpreting the change other than on the physical level. People may sometimes have sudden shifts in their intentional states, for example, in an extreme situation, which are explainable without referring to biochemical processes. In such a case, the extreme circumstances make the sudden change intelligible on an intentional level. In contrast, the situation the patient experiences in the doctor’s office while adjusting the stimulator does not make the change intelligible without reference to neural processes. The only coherent narrative available is one deprived of meaningful intentional states. Electrochemical and neurobiological events take their place.
2. Dubiel expresses how the banality of the process is humiliating to him. The fact that such a straightforward mechanism can cause deep-felt sorrows and thoughts to disappear can be troublesome. Even though complex processes are involved in DBS treatment, the causal chain seems straightforward and transparent. Simply put, DBS is a wire in your brain emitting a low current. Because of the straightforward and transparent causal chain, the explanation via biochemical processes suggests itself. The change can be explained in biochemical terms in a way that is understandable and intelligible. It is an easily available explanation that is simple to integrate into the self-narrative. The severe and instant changes can make it hard to take an intentional perspective on the change, as discussed above, and the transparency and straightforwardness of DBS facilitate the biochemical viewpoint.
3. The third feature of DBS that can pull towards a biochemical perspective is the permanent, physical presence of the implant in the head and chest. Some patients reported they feel like a robot or an electronic doll [10] or that they are “under remote control” [33]. The constant presence of the implant can visually and tangibly remind the patients of the mechanical influence they are subject to. Thereby, their behavior can appear to be guided by a mechanical device instead of intentional states and thus rather belong to the *nomological realm of law* than to the *logical space of reasons.* Meaningful, intentional actions turn into biomechanically controlled behavior. Additionally, the remote control can underline the technological accessibility of one’s characteristics. However, a recent study suggests that most patients do not notice the device very much or view it as a foreign entity [38]. For some patients, the physical presence of the implant can nevertheless be irritating.

A further issue, emerging from the diminished sense of meaning and the shift away from intentional states, is a feeling of lost control. Of course, DBS can increase the control of patients by freeing them from symptoms that reduced their control over their body and mind. However, studies have reported that some patients felt they lost control over their actions after undergoing DBS treatment [33, 38]. A variety of reasons can contribute to a feeling of lost control. One way in which DBS can contribute to a loss of control is through a shift to a biochemical view on behavior, as discussed above. Actions driven by mental states without a narrative connection to one’s past, appearing to come out of nowhere, seem out of one’s control. They are not made intelligible through the self-narrative which can make it hard to identify with them. One patient described that the “giving of power” through DBS was accompanied by a “lack of power”: *“And on the way I stopped and bought a very uncharacteristic dress, backless—completely different to what I usually do.”* [38] What made her buy the uncharacteristic dress is unintelligible to her and seemingly out of her control. By bringing forth new intentional states that lack an understandable and meaningful connection to one’s past, DBS can decrease the perception of self-control and lead to taking a biochemical view on one’s behavior.

A way to reduce the loss of meaning through DBS is by strengthening its connection to the meaningful, intentional decisions that preceded the treatment, as in the above example of a person deliberately seeking change through direct intervention. As Schechtman suggested [7], there is a route to take a different perspective on the induced changes by focusing on the choice to deal with the illness by undergoing DBS: *“After fighting all my life against depression without success, I decided to undergo DBS, through which I was freed from my depressive thoughts.”* Events playing out in the *realm of law* gain their meaning indirectly through their connection to intentional states. The DBS treatment indirectly gains meaning because it is caused by a meaningful decision. It seems that this is not only a legitimate, active way of mitigating a loss of meaning but that many people just happen to adopt this strategy to accommodate biochemical explanations into their self-narratives in a meaningful way. Moreover, because the device can be turned off it is possible to intentionally choose whether or not one wants to experience the effects of DBS in a specific situation. Thereby, the connection to intentional states is strengthened. Because DBS is generally only used for very severe disorders, switching off the device is often not a viable option. The event-specific use of direct neural interventions is therefore discussed in more detail in reference to Ritalin.

Not all patients whose personality changed through DBS acknowledge their transformation or see it as problematic. Some disagree with their families and caregivers and assure they did not change at all or see themselves as restored to their former, more energetic and thriving self after stimulation [10, 38]. The former may have an issue with the accuracy of their self-narrative. They would not experience a loss of meaning but, according to some narrative self views, they would violate a reality-constraint which would undermine their self-constitution [21] or their authenticity [27]. The latter could be cases in which DBS is understood as overriding a mental condition that plays the role of a direct, biochemical influence in the patient’s self-narrative. As argued above, this could mean that their behavior, feelings, or beliefs become more meaningful because they are no longer seen as directly influenced by a disorder such as PD. However, more empirical research would be needed to assess this claim.

### Prozac

Prozac is the brand name of an antidepressant with the active agent Fluoxetine, a selective serotonin reuptake inhibitor. It is usually taken once a day and takes approximately 3–4 weeks to build up an effect. After this period the daily dose suffices to maintain an effective amount. Prozac is very widely prescribed and can have remarkable results. However, the sometimes-astounding effects can also be unsettling. Prozac sparked a debate in neuroethics, mainly because besides brightening the mood it can also alter a person’s character [15–17, 45–48]. Through Prozac, individuals can become more outgoing, confident, or decisive. The effects of Prozac can go beyond treatment and make people “better than well” [46], raising questions about enhancement, authenticity, and other issues. The claim that Prozac can reduce meaningful emotional and personal struggles to biochemical processes has been addressed before [16]. The following adds to this debate by addressing the issue in the light of the narrative self view and by including a comparative perspective to other neural interventions. The second focus is on Prozac not only because it is widely used and debated but because it is an exemplary case of a chemical neural intervention that can cause a gradual, global change in the patient. The following discussion should apply to comparable neural interventions.

As discussed, it can be difficult to take anything but a biochemical perspective on a prompt transformation through a neural intervention because intentional states tend to change slower (with some exeptions). In contrast, Prozac leaves more room for an explanation of self-change in terms of intentional states. With the aid of Prozac, an indirect way of self-change could take part, as discussed in a fictional example by Hoffman: *“After more of my serotonin transporters were blocked, a previous deficiency in my perception of the world was corrected. This helped me to see that the world is worth living in after all.”* [18] The longer time it can take for psychoactive drugs to take effect allows for the induced changes to be partially ascribed to personal insights or changes in intentions and beliefs. Thus, slower changes can appear more meaningful and controlled. The transformation may be supported by the causal chain provoked by the drug but it need not be reduced to the biochemical process. In the example, the change of perception can deliver the content to intelligibly explain the change in intentional terms. An exclusively biochemical perspective on the process is therefore not the only option.

Moreover, the biochemical perspective is suggesting itself less than with DBS because the causal chain is less transparent and straightforward. At least from a layperson’s perspective, the causal story of Prozac and other psychopharmaceuticals is more abstract, complicated, and hidden: the active component, the molecules in the pill, are more abstract than a wire with electrodes; whereas the DBS-electrodes are directly implanted into the area where they take effect, the psychopharmaceutical is ingested and finds its way to the brain through the bloodstream; after ingestion, the causal chain of a psychopharmaceutical is hidden whereas with DBS the presence of the stimulator, which is directly connected to the brain, is constantly visible and tangible. Therefore, the biochemical account is comparatively inaccessible in the case of Prozac.

Lastly, the patient is less passive compared to DBS. Taking psychopharmaceuticals is a repeated (often daily) act, increasing the focus on the choice for the treatment and the related meaningful intentional states. Thus, even if a person integrates the transformation into the self-narrative through a biochemical explanation, it is easier to connect it to his choices, where he comes from, and where he is going to. The repeated intake represents a repeated choice for the treatment and the changes it brings about. The biochemical process at play after ingestion of the pharmaceutical is not meaningful in and of itself but the active choices and actions of the patients connected to it can be. Their active involvement can indirectly ascribe meaning, for instance, by understanding the use of a psychopharmaceutical like Prozac as a form of self-care. The gradual nature of the change, the lower transparency of the causal chain, and the patient’s active participation can make a multicausal account of the transformation, including meaningful intentional states, more appealing.

### Ritalin

Ritalin is the name of the most prescribed drug for the treatment of Attention Deficit Hyperactivity Disorder (ADHD). Ritalin can increase the attention span and make a person less distractible, impulsive, and restless. It is a short-acting form of methylphenidate—it is effective within 30 min to 1 h after ingestion and leaves the system after approximately 3–4 h [49]. Ritalin is an exemplary case of a neural intervention causing a fast, short-term change.

Because the changes induced by Ritalin are fast, as in the case with DBS, they are particularly difficult to account for in any other way but through a biochemical process. An explanation of how someone routinely changes from a hyperactive state to being focused and concentrated within 30 min after taking Ritalin in terms of that person’s intentional states would usually seem far-fetched. In most cases, it is more natural to ascribe the fast transition to the drug than to independent changes of the person’s goals, interests, and other intentional states, which do not usually occur so rapidly and without a major cause. However, some features of Ritalin and its use can soften its impact on narrative meaning-making. The short-term effect of Ritalin allows its use for event-specific purposes [50, 51]. It is possible to use it in preparation for a specific event in which additional focus and attentiveness would be helpful, such as an important exam or a social gathering. Due to the short-term effect, the change is understood in biochemical terms, but this mode of use allows for a strong connection to personal reasons and intentions. Thereby, event-specific interventions can gain meaning through a connection to other, meaningful intentional states. Moreover, the fast rise and fall of the effects of Ritalin can lead to a stronger focus on the choice for the treatment.^11^ The decision to take the drug has to be made multiple times a day. Again, this strengthens the connection of the change induced by the drug to intentional states. Most seem to be able to integrate both states on- and off-medication into a meaningful, coherent narrative, not least because with Ritalin it is possible to choose flexibly between versions of oneself for short periods of time [52].

It may appear that Ritalin ideally only affects a very specific set of characteristics, that it does not change intentions but only one’s ability to follow them through. However, it has been reported that in many cases Ritalin affects demeanor, mood, and even preferences [51]. The range of intentional states influenced by a neural intervention of course matters for the scope of a possible loss of meaning. In the case of a neural intervention which only changes very specific aspects of the individual, the loss of meaning is limited compared to one in which global characteristics are affected. In the former case, most of one’s intentional states can continually provide a meaning-generating network with an uninterrupted and intelligible history. With Ritalin, both specific and global changes are possible.

### Psychedelics

Another neural intervention, which differs in relevant aspects from other psychopharmaceuticals, is the medical use of psychedelic substances. In the last decade, the scientific interest in the use of psychedelics for medical purposes, in particular psilocybin (the active ingredient of some mushrooms) and lysergic acid diethylamide (LSD), has strongly increased. They have shown promising results for the treatment of anxiety, depression, Obsessive–Compulsive Disorder as well as addiction to tobacco and alcohol [53, 54]. Psychedelic substances can reliably induce powerful subjective experiences and altered states of consciousness. It is still inconclusive how psychedelics exert their treatment effect but the acute psychedelic experience seems to be a contributing factor [53, 55]. Psychedelics combine a direct and indirect intervention. On the one hand, the drug directly influences the brain itself on a physical level. On the other hand, the acute phenomenological experience induced by the psychedelic serves as an input into the brain, which is processed by the patient’s mental faculties. In this indirect process, both the semantic content of the psychedelic experience—what is perceived during this episode—and the psychological structure of the person that took the psychedelic are relevant. Psychedelics are an exemplary case of a neural intervention that is accompanied by an acute phenomenological experience.

Because psychedelics have an at least partly indirect effect, it is much easier to make sense of the changes they induce in terms of insights and intentional states. One could still explain the change in biochemical terms, for example, *“I came to believe that the world is worth living in after all because LSD increased the resting state connectivity between my brain areas.”* However, the changes can also straightforwardly be attributed to personal insights caused by the psychedelic experience, such as *“After I experienced a deep sense of connectedness to others, the world, and myself, I felt far less absorbed by my own issues and concerns, which helped me to see that the world is worth living in after all.”* Both perspectives on the transformation are possible, both make sense and intelligibly explain how the transformation came about. However, the latter does not lead to a loss of meaning. The self-change is understood in terms of insights and intentional states, as a meaningful response to the experience.^12^ The psychedelic experience provides content for a meaningful, narrative explanation of the change. Additionally, psychedelics are more diachronic compared to other psychoactive drugs. Their effect crucially depends on the mental state of the patient at the time of ingestion. Thereby, psychedelics directly connect to the patient’s self-narrative.

### Psychotherapy

In the case of psychotherapy (by this I mean any kind of talk therapy, for instance, cognitive therapy), the situation is altogether different from direct neural interventions. Psychotherapy works directly with intentional states to encourage self-change. It is still possible to take a biochemical perspective, for instance: *“I came to believe that the world is worth living in after all because talking about my trauma helped to increase serotonin uptake in the medial prefrontal area.”* But because intentional states are addressed directly, they offer a more straightforward explanation. The biochemical perspective does not suggest itself. I am not addressing the empirical question about whether something like an increase in serotonin uptake or, for example, increased self-knowledge is responsible for the success of psychotherapy. Psychological and biochemical processes are interconnected. They are two sides of the same coin. In the case of psychotherapy, one side of the coin tends to be in sight because this is the side that is usually directly addressed in therapy.

However, this is not always the case. Some patients in psychotherapy could benefit from knowing the biochemical basis of their disorder [57, 58]. Objectivizing and externalizing a disease can lower self-blame and make it easier to work on it, to cope with it, and to integrate it in an intelligible and meaningful self-narrative. So, in some cases, the biochemical perspective seems to be not the cause of problems but a possible solution. The patient can be freed from the need to account for a  behavior or feeling via intentional states. In line with this, it has been argued that personal bioinformation plays an instrumental role in the construction of our narrative identities [59]. If biochemical processes are integrated and connected to meaningful, intentional actions, goals, or choices a potential loss of meaning can be avoided. By embedding them in a network of intentional mental states and actions, they can become indirectly meaningful.

## Conclusion

This paper argued that a crucial and unique impact of neural interventions on the self-narrative is their possible influence on the meaning of actions, emotions, moods, and other intentional elements of the self-narrative. To uphold the coherence of the narrative, the changes neural interventions induce need to be accounted for. We can achieve this through explanations via intentional or biochemical terms, or a combination of both. Either way can uphold the coherence of the self-narrative but only explanations via intentional states directly ascribe meaning to events, actions, beliefs, feelings, etc. Neural interventions can lead to a loss of meaning because they can favor a biochemical perspective. Depriving events of meaning is not inherently negative and it can be a part of treatment. But it can be problematic, particularly if the individual is unprepared and if events are affected, he or she was not willing to have stripped of meaning. The degree to which such a pull towards a biochemical view occurs depends on the specific means of neural intervention. The examples of DBS, Prozac, Ritalin, and psychedelics identify the rate at which the change occurs, the transparency of the causal chain, how involved the patient is, and whether a phenomenological experience accompanies the change as main factors influencing the loss of meaning.

In the future, we may eventually get used to self-change through direct neural interventions such that we develop strategies that either avoid a loss of meaning or that make it less problematic. The changes induced by alcohol consumption, for instance, have been normalized to such a degree that we generally do not see them as a threat to meaning. A different question would be whether such normalization would even be desirable. Further empirical research into the self-narratives of patients could provide valuable insights about possible shifts in the narrative self-representation, their impact on meaning, and how patients cope with it. The theoretical, neurophilosophical analysis of this paper could provide a basis for studying patients’ descriptions of their treatment process. Before undergoing treatment with a direct neural intervention, patients should be informed and prepared for the possibility of a loss of meaning through a shift to a biochemical perspective. This is particularly important for treatments that highly encourage the biochemical viewpoint, notably DBS. The possibility of a loss of meaning can provide a reason to opt for psychotherapy or treatment with psychedelics over other methods, in cases where a choice is possible.

## Data Availability

Not applicable.

## Footnotes

1 Versions of a narrative self-constitution view have been introduced by Alasdair MacIntyre, Charles Taylor, Paul Riceour, Daniel Dennett, and Marya Schechtman (among others). For an overview see [19]. The argument of this paper does not depend on the specifics of how the self-narrative constitutes the self.

2 In this sense, I understand the self as a subjective, phenomenological viewpoint formed by the self-narrative. On these grounds, I reject a strictly relational account of narrative Identity, as suggested by Françoise Baylis, Mary Walker, or Catriona Mackenzie [8, 20]. Nonetheless, I do think that others crucially influence how we (can) construe our self-narrative. Moreover, the argument of this paper should be compatible with a relational view of narrative identity.

3 Intentionality is understood as the power of mental states to have content, to represent or be about things, properties, and states of affairs. It should not be confused with the ordinary meaning of the word “intention”, although I am also using intentionality in the context of intentions, reasons, and explanations for actions, which are intentional.

4 For more on hyponarrativity see [28, 29].

5 I am not entering the debate on free will or the mind–body problem. However, the questions fuelling these debates can become more pressing through direct neural interventions.

6 The biochemical perspective can also be a form of self-objectification [30].

7 For instance, whether the disease is framed as a biochemical or a psychological disorder before treatment is largely factored out.

8 I am grateful to an anonymous referee for raising this example.

9 The successful treatment of a chronic disorder by a neural intervention can sometimes cause a “burden of normality” [34]. Similar to the example of the paralyzing accident, an in and of itself meaningless process—the biochemical process of the neural intervention—causes a situation which requires intentional and meaningful adaptation. This adjustment of the patient’s self-narrative can be difficult. However, the specific challenges of the burden of normality seem to originate in disruptions of coherence and not the loss of meaning.

10 DBS can also have slower effects on mental states or it can leave mental states unaffected and only change physical aspects. The example of an abrupt change of mental states is used to illustrate how the speed of change affects meaning through narratives. It is not meant to be regarded as representative of DBS treatments.

11 However, because ADHD almost exclusively affects children and adolescents, decisions regarding the use of Ritalin are often made by parents and not the person affected by the disorder.

12 A related point has been made to suggest the use of psychedelics for moral enhancement [56].
